# Comparative Analysis of Response to Cardiac Resynchronisation Therapy Upgrades in Patients with Implantable Cardioverter-Defibrillators and Pacemakers

**DOI:** 10.3390/jcm13102755

**Published:** 2024-05-07

**Authors:** Arsalan Farhangee, Mark J. Davies, Mihai Mesina, David Roger Morgan, Benjamin J. Sieniewicz, Robyn Meyrick, Katie Gaughan, Ion Mîndrilă

**Affiliations:** 1 Department of Cardiology, Milton Keynes University Hospital, Milton Keynes MK6 5LD, UK; mark.davies@mkuh.nhs.uk; 2 Department of Cardiology, Plymouth NHS Trust Foundation, Derriford Hospital, Plymouth PL6 8DH, UK; ben.sieniewicz@nhs.net (B.J.S.); r.meyrick1@nhs.net (R.M.); 3 Department of Cardiology, United Lincolnshire NHS Trust, Lincoln County Hospital, Lincolnshire LN2 5QY, UK; david.morgan@ulh.nhs.uk (D.R.M.); katie.gaughan@ulh.nhs.uk (K.G.); 4 Department of Cardiology, Oxford University Hospital, John Radcliffe Hospital, Oxford OX3 9DU, UK; 5 Doctoral School, University of Medicine and Pharmacy of Craiova, 200349 Craiova, Romania; mesina.mihai@gmail.com (M.M.); tutu0101@yahoo.com (I.M.)

**Keywords:** cardiac resynchronisation therapy, heart failure, pacemaker, implantable cardioverter-defibrillator, QRS, left ventricular systolic dysfunction

## Abstract

**Introduction:** The efficacy of de novo cardiac resynchronisation therapy (CRT) in patients with heart failure (HF), left ventricular systolic dysfunction (LVSD), and a broad QRS morphology is well established. However, the optimal stage for upgrading patients with existing pacemakers (PPMs) or implantable cardioverter-defibrillators (ICDs) and HF with high-burden right ventricular (RV) pacing remains uncertain. Thus, this multicentre retrospective analysis compared patients with pre-existing PPMs or ICDs who underwent CRT upgrades to investigate the appropriate stage for CRT implantation in these patients and to assess the validity of treating both PPM and ICD recipients under the same recommendation level in the current guidelines. **Materials and Methods:** A total of 151 participants underwent analysis in this study, comprising 93 upgrades to cardiac resynchronisation therapy with pacemaker (CRT-P) and 58 upgrades to cardiac resynchronisation therapy with defibrillator (CRT-D) across three centres in the UK. The aim of the study was to investigate the safety and efficacy of upgrading to CRT from an existing conventional pacemaker or an ICD in the context of high-burden RV pacing. The analysis was conducted separately for each group, assessing changes in echocardiographic parameters, functional New York Heart Association (NYHA) class, and procedure-related complications. **Results:** The PPM group had a higher percentage RVP burden compared to the ICD group. Post-upgrade, NYHA functional class and EF and LV volumes improved in both groups; however, the response to an upgrade from a pacemaker was greater compared to an upgrade from an ICD. Post-procedural complication risks were similar across the two subgroups but significantly higher compared to de novo implantation. **Conclusions:** Within the CRT-P subgroup, participants exhibited better responses than their CRT-D counterparts, evident both in echocardiographic improvements and clinical outcomes. Furthermore, patients with non-ischemic cardiomyopathy (NICM) were better responders than those with ischaemic cardiomyopathy. These findings suggest that international guidelines should consider approaching each subgroup separately in the future.

## 1. Introduction

The benefits of cardiac resynchronisation therapy (CRT) implantation in patients with heart failure are well established, with demonstrated reductions in overall mortality and improvements in quality of life [1,2,3,4,5].

International guidelines clearly outline patient selection criteria for de novo CRT implantation, supported by a robust body of randomised trials. However, when it comes to upgrading an existing pacemaker implanted for bradyarrhythmia or an implantable cardioverter-defibrillator (ICD) to CRT, recommendations are primarily based on a limited number of retrospective studies. This raises concerns about the potential undertreatment of a patient population that could significantly benefit from CRT [6,7].

The 2013 European Society of Cardiology (ESC) guidelines on cardiac pacing and cardiac resynchronisation initially assigned a class IB indication for upgrading an existent pacemaker or an ICD to CRT, but this was then revised in the 2021 guidelines to a class IIa indication [8,9].

Meanwhile, the 2023 HRS/APHRS/LAHRS guidelines on cardiac physiologic pacing for the avoidance and mitigation of heart failure focuses more on physiologic pacing rather than right ventricular (RV) pacing in patients with substantial anticipated pacing needs and an LV ejection fraction of 36–50%. This can be achieved through biventricular (BiV) pacing, HIS pacing, or left bundle branch area pacing (LBBAP) to reduce the risk of pacing-induced cardiomyopathy [10]. The guidelines also recommend appropriate device optimisation to reduce high-burden RV pacing [8,9,10]. 

The BUDAPEST-CRT trial stands as the sole randomised clinical trial to date that specifically investigates CRT upgrades in patients with existing pacemakers or ICDs. Its preliminary findings lend support to the efficacy of CRT upgrades, albeit solely within the CRT-D upgrade group [11]. Aside from this trial, evidence regarding CRT upgrades stems from a collection of smaller retrospective studies.

Given that upgrading to CRT covers both conventional pacemakers and ICDs, there have only been a few small studies comparing the two. Studies on upgrading a conventional pacemaker to a CRT have been encouraging thus far; however, this is not the case for the ICD group. In a study by Vamos et al., for instance, it was demonstrated that both clinical response and long-term survival were less favourable in patients undergoing cardiac resynchronisation therapy-defibrillator (CRT-D) upgrade compared to de novo implantations [12].

Given the distinct underlying substrates between patients who have an ICD compared to those with a conventional pacemaker, we question the validity of grouping them under the same recommendation class. Instead, each group should be analysed individually. 

In this multicentre retrospective study, we evaluated patients with a conventional pacemaker implanted for bradycardia indication or post-atrioventricular (AV) node ablation with high-burden RV pacing and patients with an existent primary/secondary prevention defibrillator with high-burden RV pacing or broad QRS complex and severely impaired LV systolic function and HF who underwent an upgrade to CRT. 

## 2. Materials and Methods

### 2.1. Study Design

This multicentre retrospective study encompasses a total of 151 participants who underwent an upgrade to CRT between January 2010 and January 2020. The participants were divided into two groups: 93 upgrades to CRT-Pacemaker (CRT-P) and 58 upgrades to CRT-D. Patient data were collected from Derriford, Lincoln, and Milton Keynes Hospitals in the UK. Participants underwent their upgrade either at the time of their pacemaker generator box change or as an inpatient following admission with decompensated HF and severely impaired LV systolic dysfunction.

The aim of this study is to investigate the safety and efficacy of upgrading to CRT from an existent conventional pacemaker or an ICD in the context of high-burden RV pacing. The analysis will be conducted separately for each group, focusing on changes in echocardiographic parameters such as LVEF, LVESV, LVEDV, and LVIDd from baseline and up to 18 months post-upgrade to CRT as well as changes in functional NYHA class and procedure-related complications. 

Patients included in this study must have had an echocardiogram, pacing checks, and an electrocardiogram before the upgrade procedure, with repeated assessments 6–18 months post-upgrade, all documented in each centre’s electronic or physical database. Moreover, patients must have had, in their clinical notes, documented functional NYHA class before the upgrade procedure and 6–18 months after. 

The dataset for analysis included information from a total of 318 patients who underwent an upgrade to CRT. The main criteria for patients to be enrolled in the study were the availability of both pre- and post-upgrade echocardiographic measurements and clinical notes. Notably,167 patients were excluded from the group for various reasons. Nineteen patients were repatriated to other centres where clinical notes were not available for analysis; only six patients had a post-upgrade focused echocardiogram for the assessment of left ventricle ejection fraction, and in another 70 patients, LVEF was not assessed through Simpson’s biplane method; moreover, 11 patients had incomplete echocardiographic measurements other than LVEF; and 41 patients did not have a repeat echocardiogram after the upgrade; and six patients had an echocardiogram summarised in their clinical notes but without actual measurements and an echocardiogram report. Furthermore, there were instances where upgrade procedures failed, leading to alternative interventions. Three patients underwent an epicardial left ventricular (LV) lead implantation after a failed upgrade procedure, while another 11 patients with unsuccessful conventional upgrades ended up having a trans-septal endocardial LV lead implant. These exclusions and alternative interventions were essential considerations in ensuring the integrity and reliability of the dataset for subsequent analysis.

All 151 participants included in this study underwent echocardiographic assessments conducted by physiologists accredited by the British Society of Echocardiography. Device interrogations and optimisation were performed by a British Heart Rhythm Society-accredited physiologist. Attempts were made in all the patients to reduce high-burden RV pacing, and the decision to upgrade was either made by a consultant cardiologist or by an HF multidisciplinary team. 

The main criteria to upgrade were severely impaired LV systolic function, NYHA class, and high-burden RV pacing. A flowchart diagram of the study is available in the Supplementary Materials, Figure S1. 

### 2.2. Pacemaker Interrogation

Participants reviewed in this study underwent a pre-upgrade pacemaker/ICD interrogation, which provided information on the percentage of RV pacing. Physical notes were analysed in those without electronic records to obtain the necessary information. A post-implant CRT interrogation was performed in all patients to determine the percentage of BiV pacing.

From the time of the initial implantation of an antibody pacemaker or an ICD, patients were followed up every 6–12 months. Device optimisation was performed in all the patients, both before and after the procedure, to ensure that adequate attempts were made to limit high-burden RV pacing. Moreover, following an upgrade, optimisation was performed to maintain high-burden BiV pacing, thus ensuring effective CRT.

### 2.3. Echocardiography

We noted that a routine follow-up echocardiogram was not routinely requested for all the patients, with the decision to request one being left to the discretion of the overseeing physician. The typical pattern for requesting a follow-up echocardiogram was at least six months post-procedure. However, variations occurred, with some physicians opting for an annual echocardiogram, and in a few cases, the follow-up echocardiogram being scheduled at 18 months post-implantation. Hence, a period of 6–12 months post-implant follow-up period was set for our study.

Various parameters, including LVEF (assessed by Simpson’s biplane), left ventricle end-diastolic volume (LVEDV), left ventricle end-systolic volume (LVESV), and left ventricle internal dimension in diastole (LVIDd) were analysed based on international chamber quantification guidelines [12] before and after the upgrade. Participants with incomplete echocardiographic assessments were excluded from the study. In summary, the study involved 318 participants. Each case was analysed for a second time by an experienced cardiac physiologist and measurements such as left ventricle ejection fraction (LVEF), left ventricle end-diastolic volume (LVEDV) and left ventricle end-systolic volume (LVESV) were repeated manually. The strength of agreement of intraobserver analysis for a cut-off point of 5% was accepted and included in the study. We noted that the variation was the highest for visual estimation of the ejection fraction and lowest when using the Simpson biplane, which also had the highest interobserver agreement.

Patient characteristics, including aetiology of LV dysfunction, NYHA functional classification, medication history, associated comorbidities, and post-upgrade complications were documented from their electronic and/or physical medical records.

The distribution of patients based on the aetiology of cardiomyopathy, medication history, and associated comorbidities are listed in Table 1, for detailed information, see the Supplementary Materials, Table S1.

### 2.4. Statistical Analysis

Statistical analysis was performed using Microsoft Excel (Microsoft Corp., Redmond, WA, USA), together with the XLSTAT add-on for MS Excel (Addinsoft SARL, Paris, France). The descriptive analysis of the study group was performed with Excel (Microsoft Corp., Redmond, WA, USA), while complex statistical analysis (Kruskal–Wallis test and Post Hoc Mann–Whitney U test using a Bonferroni correction) having set the significance level at 0.05 was performed using XLSTAT add-on for MS Excel (Addinsoft SARL, Paris, France). Normality tests (Anderson–Darling) and complex statistical tests (chi-squared, Mann–Whitney–Wilcoxon) were performed using XLSTAT.

Since most of the numerical variables recorded in our study deviated from a normal (Gaussian) distribution, the nonparametric Mann–Whitney test was primarily used to detect significant differences between the values in the compared data series for patient groups. *p*-value < 0.05 was considered statistically significant.

Additionally, multivariate linear regression was used to compare the effects of aetiology, medication history, percentage RVP, and comorbidities on post-upgrade NYHA and EF change. 

## 3. Results

### 3.1. Analysis of Upgrade to CRT-P Participants

A total of 93 patients were upgraded from a conventional pacemaker to CRT. This study population comprised 64 males and 29 females, with a median age of 82 ± 10 years.

The mean pre-upgrade QRS duration was 181 ± 21 ms, compared to 114 ± 15 ms after the upgrade, indicating a narrower QRS duration of at least 66 ± 25 ms (*p* value < 0.0001). The pre-upgrade LVESV was 121 ± 33 mL, compared to 84 ± 33 mL after the upgrade with a post-upgrade decrease in LVESV of 36 ± 24 mL (*p* value of 0.0001). The pre-upgrade LVIDd measured in 2M-mode was 5.6 ± 0.7 cm, compared to 5.1 ± 0.7 cm after the upgrade, showing a decrease in LVIDd of 0.5 ± 0.4 cm (*p* value of 0.0011). The pre-upgrade mean LVEDV was 170 ± 50 mL and post-upgrade LVEDV was 128 ± 46 mL, with a median decrease in LVEDV of 41 ± 29 mL (*p* value of 0.0003). The mean LVEF before the upgrade was 30 ± 9% and 43 ± 11% after the upgrade, demonstrating an increase in LVEF of 12 ± 9% (*p* value of 0.0002). The mean NYHA class before upgrade was 2.88 compared to a post-upgrade NYHA class of 1.71, indicating at least a one-grade classification decrease in NYHA class, which was statistically significant with a *p* value of <0.0001.

Patients who were prescribed an angiotensin receptor–neprilysin inhibitor (ARNi) exhibited a better LVEF before upgrade of 34 ± 7% compared to 26 ± 10% in those without ARNi and responded better (46 ± 9%) compared to those without ARNi (40 ± 11%), which was statistically significant with a *p* value of 0.0053.

Patients who were prescribed an SGLT-2 inhibitor had a better LVEF before upgrade but there was no statistical difference. Similarly, no statistically significant differences were observed in pre-upgrade LVEF or post-upgrade LVEF improvement among patients who were prescribed a mineral receptor antagonist (MRA) or a beta-blocker.

Patients with ischaemic heart disease had a similar pre-upgrade NYHA classification compared to those with non-ischaemic aetiology; however, patients with non-ischaemic cardiomyopathy had a greater decrease in NYHA class compared to those with ischaemic cardiomyopathy. Moreover, there was a greater increase in EF in patients with non-ischaemic heart disease compared to those with ischaemic heart disease, but this was not statistically significant.

### 3.2. Analysis of Upgrade to CRT-D Participants

A total of 58 patients underwent CRT-D upgrade, with a predominantly male population (72%) and a median age of 76 ± 10 years.

There was no statistically significant difference between patients in sinus rhythm compared to those with atrial arrhythmia. As anticipated, ischemic cardiomyopathy was the predominant aetiology (72%). The mean pre-upgrade QRS duration was 170 ± 25 ms, compared to that after upgrade, which was 117 ± 12 ms, indicating a narrower QRS duration of at least 52 ± 25 ms. Pre-upgrade LVESV was 151 ± 47 mL, and after upgrade, it was 128 ± 58 mL, with a post-upgrade decrease in LVESV of 22 ± 32 mL. Pre-upgrade LVIDd measured in 2M-mode was 6.3 ± 0.9 cm compared to 5.9 ± 1 cm after upgrade, showing a decrease in LVIDd of 0.38 ± 0.53 cm. The pre-upgrade mean LVEDV was 220 ± 69 mL and the post-upgrade LVEDV was 187 ± 81 mL, with a median decrease in LVEDV of 32 ± 56 mL. The mean LVEF before upgrade was 23 ± 11% and post-upgrade LVEF was 33 ± 13%, demonstrating an increase in LVEF of 10 ± 14%. The mean NYHA class before upgrade was 3.1 compared to a post-upgrade NYHA class of 2, indicating at least a one-grade classification decrease in NYHA class. The improvements in all the analysed parameters were statistically significant.

Patients treated with ARNi exhibited a superior pre-upgrade NYHA class but did not experience significant echocardiographic improvement following the upgrade. On the other hand, patients treated with SGLT-2i demonstrated a statistically significant improvement in LVIDd after the upgrade and achieved a better NYHA class, while patients treated with MRA experienced a superior post-upgrade LVESV decrease, a better NYHA class, and an improvement in EF.

### 3.3. Comparative Analysis of Patients with CRTP Upgrade vs. CRTD Upgrade

The sex ratio was similar in both groups, close to 70% in favour of male patients. Patients with an upgrade to ICD were younger (median 76 ± 10 years compared to 82 ± 10 years in the CRT-P group). As expected, there was a high prevalence of patients with ischaemic cardiomyopathy in the ICD group, with a higher prevalence of non-ischaemic cardiomyopathy in PPM participants. The use of heart failure medication was similar between the two groups. Patients in the ICD group had a higher prevalence of diabetes compared to those in the PPM group. 

The PPM group had a higher percentage of RVP compared to the ICD group. A statistically significant difference was observed between those with RVP > 40% and those with <40% RVP, with a multivariate linear regression analysis demonstrating a greater post-upgrade increase in EF in patients with RVP > 40% compared to those with RVP < 40% (44.34 ± 10.57 versus 35.73 ± 13.94%). 

Post-upgrade LVIDd and LVEDV reductions in patients with a high percentage of RVP (>40%) were higher compared to those with <40% RVP.

**Figure 1:**
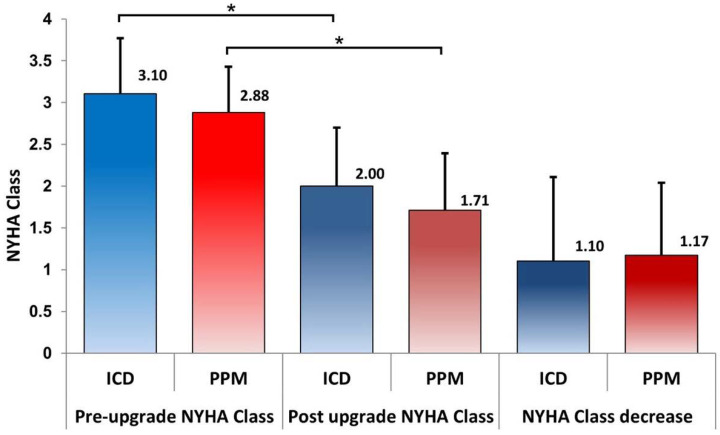
Patients in the ICD group had a higher NYHA class pre-upgrade compared to those with a PPM, with an almost identical reduction post-upgrade in both groups (Bonferroni Mann–Whitney U test; * *p* < 0.05).

Patients in the ICD group were in a higher NYHA class before upgrade compared to the PPM group, and there was a significant improvement in both groups, as illustrated in Figure 1.

### 3.4. Analysis of Echocardiographic Parameters

**Figure 2:**
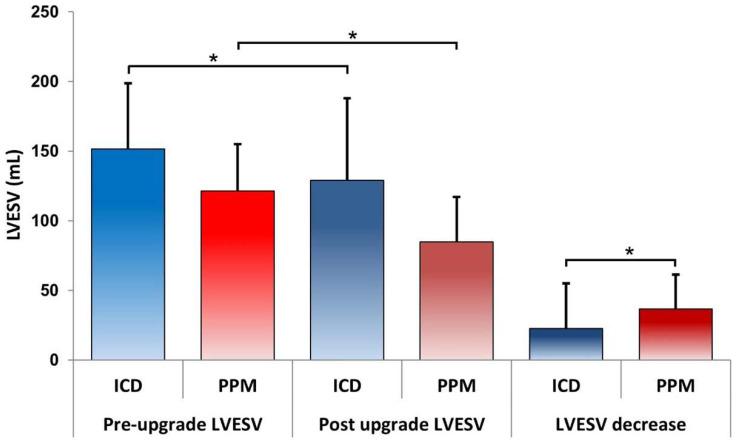
The figure illustrates a comparison of pre- and post-upgrade LVESV in ICD and PPM patients. Statistically significant differences were observed between the pre- and post-upgrade volumes in both groups, with ICD patients consistently exhibiting higher values. Additionally, a comparison of the reduction in LVESV revealed a significantly greater decrease in patients with PPM (*p* Mann–Whitney = 0.019 < 0.05), suggesting a superior recovery in this group (Bonferroni Mann–Whitney U test; * *p* < 0.05).

Prior to the upgrade, the LVESV was significantly higher in the ICD group compared to the PPM group. Additionally, participants in the PPM group experienced a significantly greater reduction in LVESV following the upgrade compared to those in the ICD group (22 ± 32 mL in the CRT-D group versus 36 ± 24 mL in the CRT-P group, *p* = 0.019). This finding suggests a superior response to the upgrade in the PPM group, as illustrated in Figure 2. 

The pre-upgrade LVIDd in patients with ICDs was higher than in those with PPM; however, although in both groups there was a significant reduction in LVIDd after the upgrade, the direct comparison between the two groups was not significant (*p* = 0.123).

**Figure 3:**
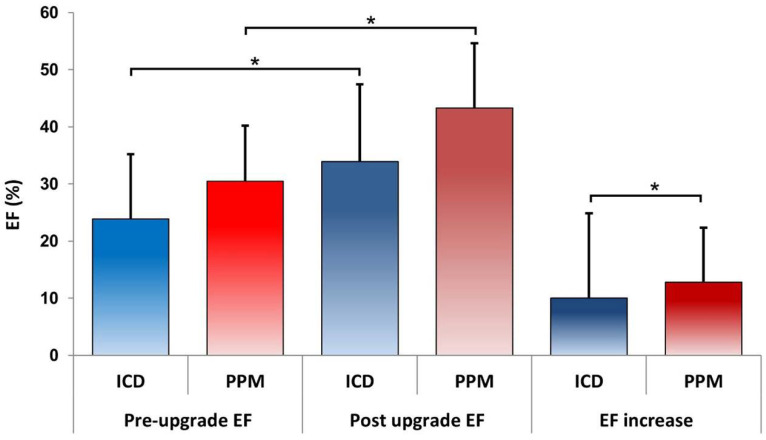
EF was greater in patients with PPM, with a highly significant level of confidence (*p* < 0.001) both pre- and post-upgrade. Moreover, the increase in EF percentage was significantly greater in patients with PPM (Bonferroni Mann–Whitney U test; * *p* <0.05).

Finally, EF was significantly lower in the ICD group before upgrade compared to that in the PPM group (23 ± 11% versus 30 ± 9), and the post-upgrade increase in patients with CRT-P was significantly higher compared to that in the CRT-D group (10 ± 14 versus 12.8 ± 9.53), *p* = 0.032 as illustrated in Figure 3.

For detailed information on the components of the study outcome, please refer to Supplementary Materials, Figures S2–S9.

### 3.5. Complications

**Figure 4:**
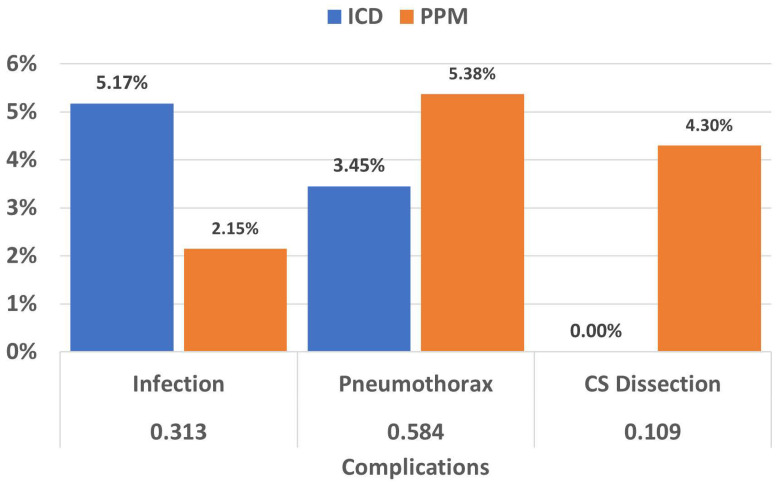
While there were numerical differences between the occurrences of infection (5.17% versus 2.15%), pneumothorax (3.45% versus 5.38%), and CS dissection (0% versus 4.30%), these differences were not statistically significant. In all three cases, the chi-squared *p*-value was greater than 0.05.

In regard to post-upgrade complications, three upgrades in the ICD group (5.17%) were complicated by infection compared to two in the PPM group (2.15%). Two cases of pneumothorax occurred in CRT-D participants (3.45%) compared to five in the PPM group (5.38%). There were no cases of cardiac tamponade due to coronary sinus (CS) dissection in the ICD group compared to four in the CRT-P group (4.30%). Fourteen cases experienced unsuccessful upgrades via the coronary sinus: one due to persistent phrenic nerve stimulation, five due to high pacing thresholds, and eight due to difficulty cannulating the coronary sinus or inability to identify a suitable side branch. Eleven patients ended up having trans-septal LV endocardial lead placement (seven CRT-D and four CRT-P), and three patients received surgical epicardial LV lead. The rates of complications are illustrated in Figure 4.

## 4. Discussion

To date, this observational study represents the most comprehensive analysis of patients with pacing-induced cardiomyopathy who have undergone an upgrade to CRT either from a pacemaker or an ICD. Left ventricle remodelling is a recognised complication of chronic RVP. For instance, in a study by Fang et al. involving 93 patients, it was revealed that half of the patients would develop heart failure (HF) and impaired systolic function due to high-burden RVP [13]. The BLOCK-HF trial demonstrated that, in patients with atrioventricular block and systolic dysfunction, BiV pacing not only reduces the risk of mortality/morbidity but also leads to better clinical outcomes, including improved quality of life and HF status, compared to RV pacing [14]. Furthermore, several other studies have demonstrated that, in patients with HF and chronic RVP, upgrade to CRT was similar to de novo CRT implantation in the long term in terms of mortality, reverse LV remodelling and symptomatic improvement [15,16,17,18,19,20]. Patient selection is key in upgrading groups, particularly in regard to upgrading an ICD to CRTD. The benefits of CRT-D are clear in patients with ischemic cardiomyopathy, severely impaired LV, and a broad QRS complex. However, in patients with non-ischemic cardiomyopathy, several studies suggest that the mortality benefit of a CRT-D is only present in the short term and attenuates over time [21]. This hypothesis is also confirmed by the DANISH trial, which demonstrated that prophylactic ICD implantation in patients with non-ischaemic cardiomyopathy (NICM) was not associated with a significantly lower long-term mortality [22,23]. On the other hand, the results from the BUDAPEST-CRT trial suggest that an upgrade to CRT-D in patients with LVEF < 35%, a wide paced QRS complex, and high-burden RV pacing (more than 20%) reduces HF hospitalisation and all-cause mortality while improving LV function [11]. Currently, international guidelines approach both sub-categories under the same class and level of recommendation; the 2021 European Society of Cardiology (ESC) guidelines on cardiac pacing and cardiac resynchronisation recommend cardiac resynchronisation therapy in patients with a high anticipated burden of RV pacing and impaired LV and those undergoing AVJ ablation regardless of their aetiology. Similarly, the 2023 HRS/APHRS/LAHRS guidelines on cardiac physiologic pacing for the avoidance and mitigation of heart failure recommends physiologic pacing achieved through biventricular pacing or conduction system pacing rather than right ventricular (RV) pacing in patients with anticipated RV pacing burden and an impaired LV ejection fraction. It clearly demonstrates the importance of LV ventricle remodelling due to chronic RV pacing in patients with heart failure and impaired LV function and the benefit of upgrading this group of patients to a CRT; however, the current guidelines fail to cover response to CRT in various forms of cardiomyopathies, in particular, those with ischaemic aetiology. This suggests that the decision-making process in this group of patients is relatively complex compared to those with an upgrade to CRT-P. Thus, it would be reasonable to assume that due to the intricate nature of upgrading to a CRT-D compared to a CRTP, a separate approach by the guidelines for each group would significantly add value in terms of safe decision making and timely identification of those groups of patients who would benefit more. 

Our study reveals that patients in the PPM group exhibited a greater response to CRT upgrade compared to those in the ICD group. This observation aligns with the findings of other trials involving patients with non-ischemic dilated cardiomyopathy (DCM), who seem to demonstrate an improved response to CRT driven by left bundle branch block (LBBB) electrical desynchrony over and above a poor underlying substrate. It also demonstrates that patients with non-ischaemic cardiomyopathy are better responders than those with ischaemic aetiology. This may indicate that pure electrical desynchrony is more likely to respond to CRT if it demonstrates more native activation, supporting the rationale for ongoing trials comparing CRT to conduction system pacing. Multiple studies have demonstrated the role of conduction system pacing as a suitable alternative for this group of patients, and in a study by Lustgarten et al., HBP was found to have an equivalent CRT response [24]. These studies are in line with multiple other studies demonstrating that conduction system pacing is an effective and feasible approach and can have similar outcomes as in CRT [25,26,27,28,29]. As chronic RV pacing cardiomyopathy patients have no intrinsic muscle disease, one would predict that they are more likely to be responsive to effective CRT. In patients with NICM, cardiac magnetic resonance imaging (CMR) can be used as a guide to determine whether an upgrade to CRTP or CRTD is more likely to be beneficial, with studies suggesting that the presence of left ventricular scar burden is associated with all-cause mortality, cardiovascular mortality, ventricular tachyarrhythmia, and sudden cardiac death [30]. The study also demonstrates that upgrades to BiV pacing have a higher risk of complications compared to de novo implantation. This finding undermines the findings of a study by Pothineni et al. [31] and a survey by Bogale et al., comparing the outcomes between de novo implants with upgrades to CRT [32], and indicating no significant difference in regard to complications between the two groups.

Optimal medical therapy, in conjunction with device therapy, has been shown to improve prognosis and lead to reverse remodelling in patients with heart failure. The PROVE-HF trial demonstrated that Sacubitril/Valsartan, a combination of an angiotensin receptor blocker (ARB) and a neprilysin inhibitor (NEP inhibitor), improved NTproBNP levels and LV volumes at 12 months in patients with HFrEF, providing a reverse cardiac remodelling effect [33]. SGLT-2 inhibitors, the latest addition to heart failure treatment, have been shown to reduce the rate of hospitalisation and morbidity [34].

Our study highlights the benefits of contemporary heart failure medical therapy in conjunction with device therapy. Patients with ARNi therapy had a better ejection fraction (EF) before upgrade in those with non-ischemic cardiomyopathy and experienced a greater increase in EF compared to those without ARNi. This observation was evident in both the CRTP and CRTD upgrade groups. Additionally, patients who were on an SGLT-2 inhibitor had a greater reduction in LVIDd post-upgrade compared to those without an SGLT-2 inhibitor. In the CRT-D group, patients who were on an MRA demonstrated a greater improvement in post-upgrade EF, NYHA class, and LVESV. These findings demonstrate that contemporary heart failure medical therapy alongside device therapy provides new insights into myocardial reverse remodelling, which in some patients may lead to myocardial recovery and remission. This makes a case for goal-directed therapy and the advancement of synergic medical optimisation in conjunction with CRT.

In summary, this study demonstrates the presence of adverse LV remodelling due to chronic RVP. Upgrading to CRT improved EF, reduced LVESV, LVEDV and LVIDd, and led to a significant improvement in clinical NYHA class in both groups, with a particularly high improvement in CRT-P. These findings coincide with those of a systematic review and meta-analysis performed by Kaza et al. in patients who had an upgrade to CRT [35,36]. However, upgrading to CRT also led to substantial post-procedural complications. This underscores the importance of patient selection and the implementation of multidisciplinary approaches in determining the appropriateness of upgrading to CRT.

This study also highlights and supports the rationale and need for ongoing trials comparing responses to upgrades to CRT in various forms of cardiomyopathy but also whether we should routinely implant a CRT in all patients with AV block who require a CIED implant. 

## 5. Conclusions

Post-procedural complication risks were similar across the two subgroups but significantly higher compared to de novo implantation. The CRTP subgroup were better responders than the CRTD subgroup both from an echocardiographic as well as a clinical perspective. Furthermore, patients with NICM demonstrated superior responsiveness compared to those with ischemic cardiomyopathy. Within the pacemaker group, there was a higher prevalence of non-ischemic cardiomyopathy with a higher burden of right ventricular pacing compared to the ICD group, and an upgrade to CRT exhibited a greater clinical response and an improvement in echocardiographic parameters. This may indicate that pure electrical desynchrony is more likely to respond to CRT. Furthermore, within the ICD group, there was a higher prevalence of ischemic cardiomyopathy and inferior RV pacing burden compared to the PPM group. This explains the higher rate of pacing-induced cardiomyopathy within the PPM group. These findings suggest that current international guidelines should consider a more differentiated approach for each subgroup in the future.

## 6. Limitations

In spite of the valuable insights it offers, it should also be acknowledged that this study exhibits certain limitations. For instance, the study relied solely on clinical and echocardiographic assessments of patients before and after the upgrade, limiting the scope of analyses. Moreover, brain natriuretic peptide (BNP) measurements, which could have provided valuable insights, were not routinely collected for most patients before and after CRT upgrade, although the available data on LV reverse remodelling are robust, there is clear evidence in the literature that significant lowering of BNP levels is associated with a greater improvement in left ventricle ejection fraction and the reduction in systolic and diastolic volumes. Similarly, a 6 min walk test, a valuable measure of functional capacity, could be useful in predicting echocardiographic reverse remodelling and long-term clinical outcomes in patients with heart failure receiving a CRT. However, neither BNP levels nor 6 min walk tests (6MWT) are routinely performed before or after a CRT and their role is limited only for research purposes.

Furthermore, the study is limited by its non-randomised design, and we were unable to delineate the effect specific to different cardiomyopathies and their response to CRT since a group of patients with LVSD had a mixed aetiology. Additionally, the variable follow-up duration, ranging from 6 to 8 months, complicated the comparisons we made between groups. 

Another concern is the slightly higher post-procedural pneumothorax rates observed in our study compared to other studies. This may be attributable to the predominant use of subclavian approaches in two of the three centres. However, a larger sample size is needed to definitively determine whether this association reflects a true trend or is simply a small sample size anomaly.

Finally, superior vena cava (SVC) occlusion is a frequently encountered complication associated with device upgrades. However, a comprehensive analysis of these complications was not feasible due to the lack of detailed information in post-procedure documentation.

## Figures and Tables

**Table 1:** Demographics for CRT-P and CRT-D upgrade subgroups.

	All Patients	Patients with an Upgrade to CRT-P	Patients with an Upgrade to CRT-D	*p*-Value Chi-Square
Age (years)	79 ± 10 (151)	82 ± 10 (93)	76 ± 10 (58)	0.638372271
Sex				0.45816477
Female	45 (30%)	64 (69%)	42 (73%)	
Male	106 (70%)	29 (31%)	16 (27%)	
Rhythm				0.45816477
Sinus rhythm	47 (31%)	62 (66%)	42 (73%)	
Atrial arrhythmia	104 (69%)	31 (34%)	16 (27%)	
Aetiology				
IHD	77 (51%)	35 (37.7%)	42 (72.4%)	<0.0001
Non-IHD	74 (49%)	58 (62.3%)	16 (28.6%)	<0.0001
HF Medications				
Beta-blockers	151 (100%)	93 (100%)	58 (100%)	0.312787433
MRA	123 (80%)	79 (85%)	44 (75%)	0.162412502
ARNi	82 (55%)	46 (49.4%)	36 (62%)	0.13039338
SGLT-2	77 (51.5%)	43 (46%)	33 (57%)	0.202565706
Comorbidities				
Diabetes	62 (24.7%)	33 (35.4%)	29 (50%)	0.07779673
Hypertension	132 (85%)	83 (85.9%)	49 (84.5%)	0.390548717
CKD				0.060271208
	65 (42.5%)	42 (45%)	23 (40%)	
	53 (34.7%)	34 (36.5%)	19 (33%)	
	12 (8.5%)	4 (4.3%)	8 (13%)	
	12 (8.2%)	6 (6.5)	6 (10%)	

IHD: ischaemic heart disease; non-IHD: non-ischaemic heart disease; HF: heart failure; MRA: mineralocorticoid receptor antagonists; ARNi: angiotensin receptor–neprilysin inhibitor; and SGLT-2: sodium–glucose transport protein 2 inhibitors; CKD: Chronic Kidney Disease.

## Data Availability

The datasets generated and/or analyzed during the current study are available from the corresponding author upon request.

## Supplementary Materials

The following supporting information can be downloaded at: https://www.mdpi.com/article/10.3390/jcm13102755/s1, Figure S1. Flow chart diagram. Figure S2. Patients in the PPM group had significantly greater QRS duration compared to ICD group pre-upgrade to CRT (p Mann Whitney = 0.007 < 0.05). Post upgrade reduction in QRS duration was statistically significant within the PPM group (*p* = 0.003 < 0.05). Figure S3: Comparing LVESV, there were highly significant differences between the pre- and post-upgrade volumes measured in the ICD and PPM patients, in both situations ICD patients having greater values. Comparing the reduction in LVESV, we noticed a significant reduction of LVESV within the PPM group compared to the ICD group(p Mann-Whitney = 0.019 < 0.05). Figure S4: The ICD group had a greater LVIDd both, pre-and post-upgrade however, the post-upgrade reduction in LVIDd was not statistically significant comparing the two groups (*p* = 0.123 > 0.05). Figure S5: LVEDV was significantly greater in patients with ICD, both pre- and post-upgrade (*p* < 0.001). Post upgrade, the PPM group had a greater reduction, however statistically not significant when compared to the ICD group (p Mann-Whitney *p* = 0.593). Figure S6: High prevalence of IHD in the ICD group (p Chi square < 0.001). High prevalence of non-ischemic DCM in PPM patients. Figure S7: Patients within the ICD group had an increased prevalence of diabetes compared to the PPM group; however, the difference was not statistically significant (p Chi square = 0.078 > 0.05). Hypertension prevalence rates were similar in the two subgroups. Figure S8: There were no significant differences in the use of the four pillars of heart failure treatment” between the ICD and PPM groups. Figure S9: The prevalence of chronic kidney disease within the two subgroups were non-significant (p Chi square = 0.189 > 0.05). Table S1: Baseline and post upgrade characteristics.
