# Supplementary material for: Comparative Analysis of Response to Cardiac Resynchronisation Therapy Upgrades in Patients with Implantable Cardioverter-Defibrillators and Pacemakers
Source: J Clin Med. 2024 May 7;13(10):2755. doi: 10.3390/jcm13102755 (PMC11122322; doi:10.3390/jcm13102755)
Supplement: Supplementary file 1 [file jcm-13-02755-s001.zip › jcm-2966620-supplementary.pdf]

Supplementary Appendix.

1. Supplementary Figure 1. Consort Diagram – page 1
2. Baseline and post upgrade characteristics- Page 2
3. Components of the study outcome – page 3

Figure S1. Flow chart diagram.

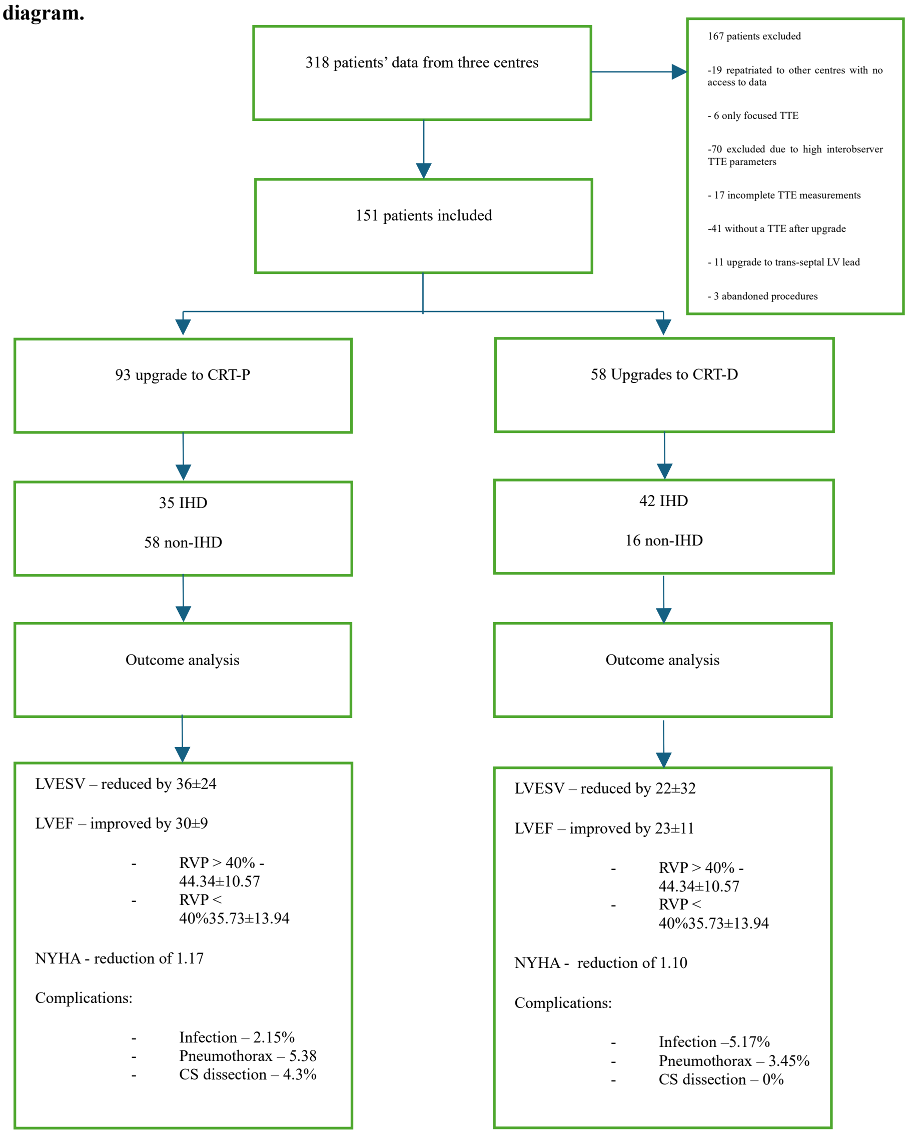

Table S1: Baseline and post upgrade characteristics:

Variable	Observations	Obs. without missing data	Minimum	Maximum	Mean	Std. deviation	Variable\Test	Mann-Whitney
Pre-upgrade QRSD | ICD	58	58	123	221	170.52	25.46	Pre-upgrade QRSD	**0.0073**
Pre-upgrade QRSD | PPM Upgrade	93	93	137	230	181.56	21.60		
Post upgrade QRSD | ICD	58	58	90	144	117.69	12.19	Post upgrade QRSD	0.3244
Post upgrade QRSD | PPM Upgrade	93	93	35	168	114.88	15.19		
QRSD decrease | ICD	58	58	-13	108	52.83	25.46	QRSD reduction	**0.0029**
QRSD decrease | PPM Upgrade	93	93	15	165	66.68	25.48		
Pre upgrade LVESV (biplane) | ICD	58	58	74	336	151.66	47.03	Pre upgrade LVESV (biplane)	**< 0,0001**
Pre upgrade LVESV (biplane) | PPM Upgrade	93	93	45	223	121.38	33.56		
Post upgrade LVESV (Biplane) | ICD	58	58	49	267	128.98	58.93	Post upgrade LVESV (Biplane)	**< 0,0001**
Post upgrade LVESV (Biplane) | PPM Upgrade	93	93	35	193	84.77	32.44		
LVESV decrease | ICD	58	58	-70	125	22.67	32.33	LVESV reduction	**0.0192**
LVESV decrease | PPM Upgrade	93	93	-11	118	36.60	24.69		
Pre upgrade LVIDd (M Mode) | ICD	58	58	4.4	8.6	6.32	0.89	Pre upgrade LVIDd (M Mode)	**< 0,0001**
Pre upgrade LVIDd (M Mode) | PPM Upgrade	93	93	4.3	7.7	5.63	0.72		
Post upgrade LVIDd (M Mode) | ICD	58	58	3.9	8.7	5.94	1.08	Post upgrade LVIDd (M Mode)	**< 0,0001**
Post upgrade LVIDd (M Mode) | PPM Upgrade	93	93	3	7.2	5.10	0.80		
LVIDd decrease | ICD	58	58	-1.5	1.6	0.38	0.69	LVIDd reduction	0.1229
LVIDd decrease | PPM Upgrade	93	93	-0.6	2.05	0.53	0.47		
Pre upgrade EF (simpson biplane) | ICD	58	58	10	55	23.88	11.33	Pre upgrade EF (simpson biplane)	**< 0,0001**
Pre upgrade EF (simpson biplane) | PPM Upgrade	93	93	9	55	30.52	9.70		
Post upgrade EF (Simpson biplane) | ICD	58	58	10	62	33.90	13.56	Post upgrade EF (Simpson biplane)	**< 0,0001**
Post upgrade EF (Simpson biplane) | PPM Upgrade	93	93	10	65	43.32	11.29		
EF increase | ICD	58	58	-18	45	10.02	14.85	EF increase	**0.0322**
EF increase | PPM Upgrade	93	93	-10	40	12.81	9.53		
Pre upgrade NYHA Class | ICD	58	58	2	4	3.10	0.67	Pre upgrade NYHA Class	**0.0347**
Pre upgrade NYHA Class | PPM Upgrade	93	93	1	4	2.88	0.55		
0-18 Month post upgrade NYHA class | ICD	58	58	1	3	2.00	0.70	0-18 Month post upgrade NYHA class	**0.0134**
0-18 Month post upgrade NYHA class | PPM Upgrade	93	93	1	3	1.71	0.68		
NYHA decrease | ICD	58	58	0	3	1.10	1.00	NYHA reduction	0.5373
NYHA decrease | PPM Upgrade	93	93	-1	3	1.17	0.87		
Pre upgrade LEDV (Biplane) | ICD	58	58	123	400	219.91	69.05	Pre upgrade LEDV (Biplane)	**< 0,0001**
Pre upgrade LEDV (Biplane) | PPM Upgrade	93	93	81	349	170.18	50.54		
Post upgrade LEDV (Biplane) | ICD	58	58	66	421	187.22	81.37	Post upgrade LEDV (Biplane)	**< 0,0001**
Post upgrade LEDV (Biplane) | PPM Upgrade	93	93	62	275	128.95	46.86		
LEDV decrease | ICD	58	58	-117	141	32.70	56.56	LEDV reduction	0.5935
LEDV decrease | PPM Upgrade	93	93	-12	151	41.24	29.74		

Components of the study outcome:

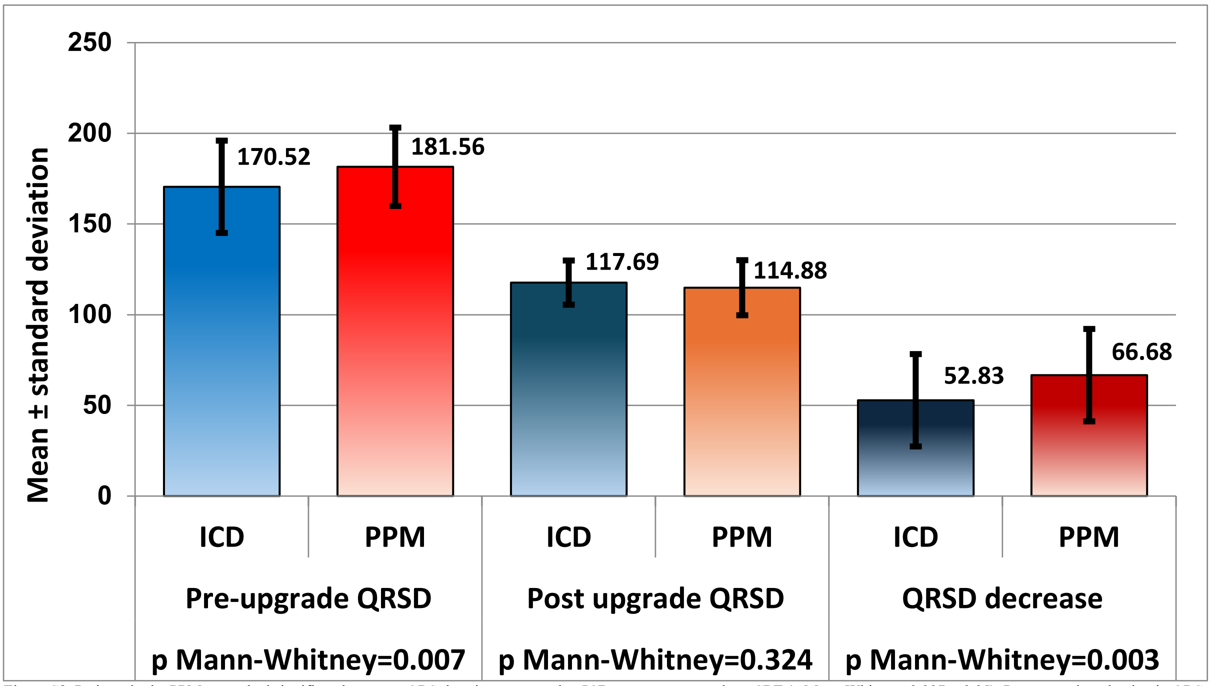

Figure S2. Patients in the PPM group had significantly greater QRS duration compared to ICD group pre-upgrade to CRT (p Mann Whitney=0.007 < 0,05). Post upgrade reduction in QRS duration was statistically significant within the PPM group (p=0.003<0.05).

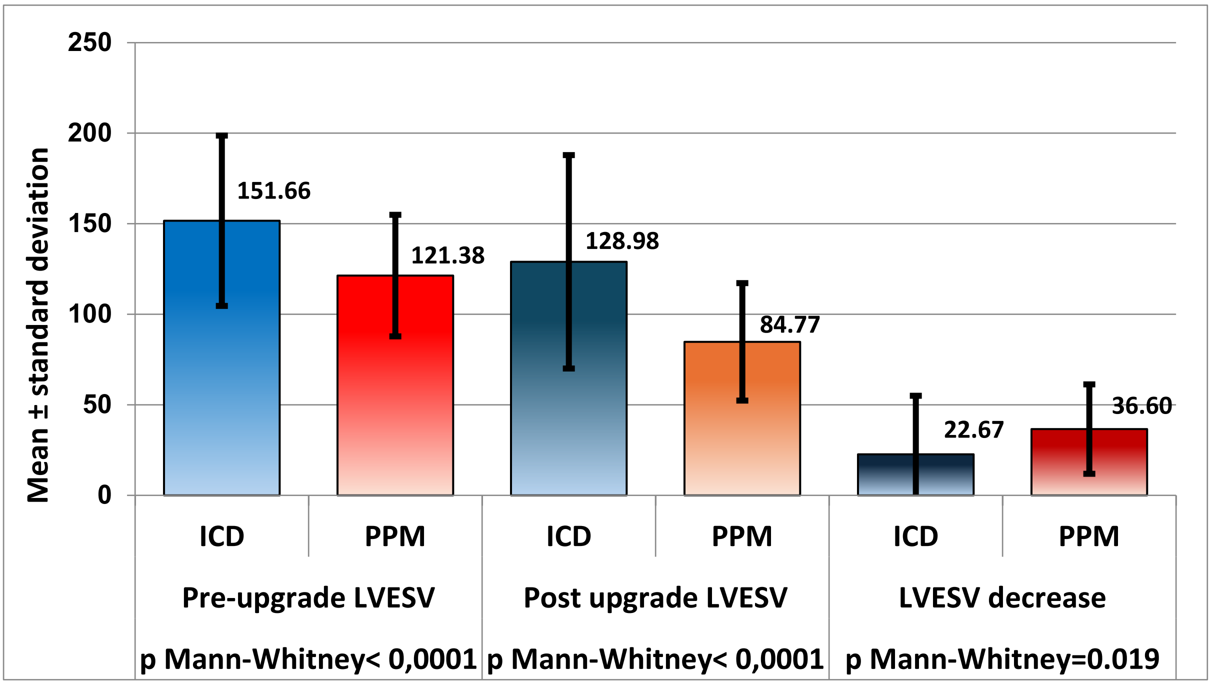

Figure S3: Comparing LVESV, there were highly significant differences between the pre- and post-upgrade volumes measured in the ICD and PPM patients, in both situations ICD patients having greater values. Comparing the reduction in LVESV, we noticed a significant reduction of LVESV within the PPM group compared to the ICD group(p Mann-Whitney=0.019<0.05).

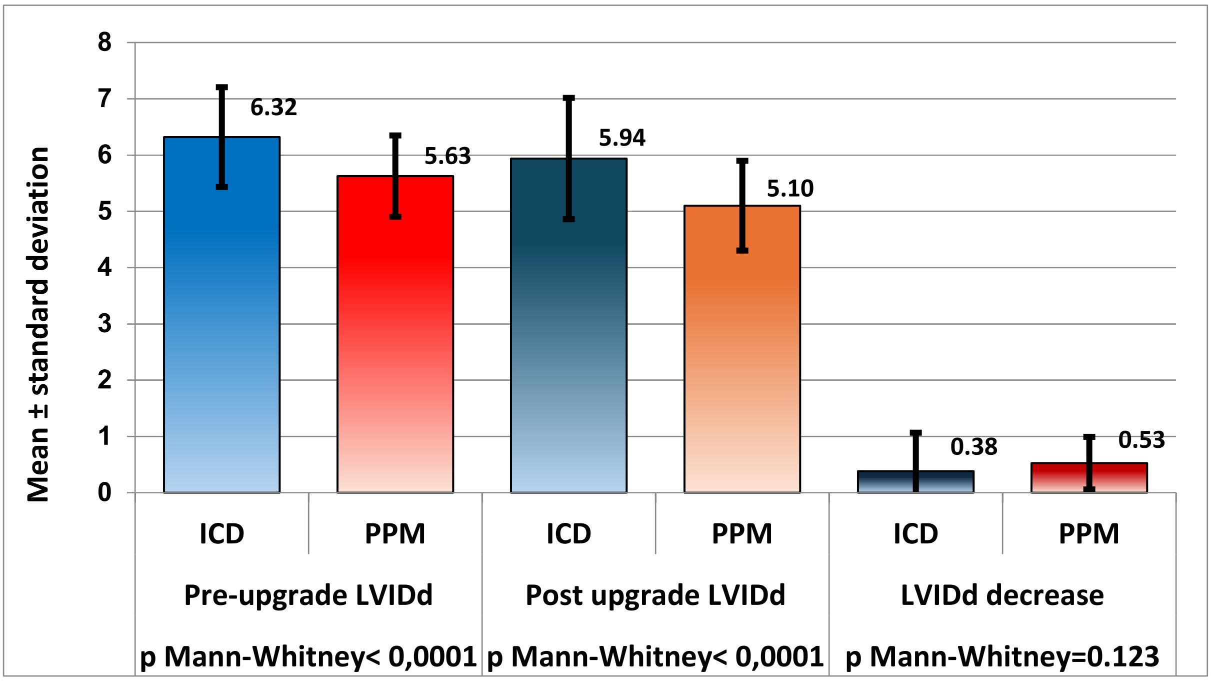

Figure S4: The ICD group had a greater LVIDd both, pre-and post-upgrade however, the post-upgrade reduction in LVIDd was not statistically significant comparing the two groups ($p=0.123>0.05$).

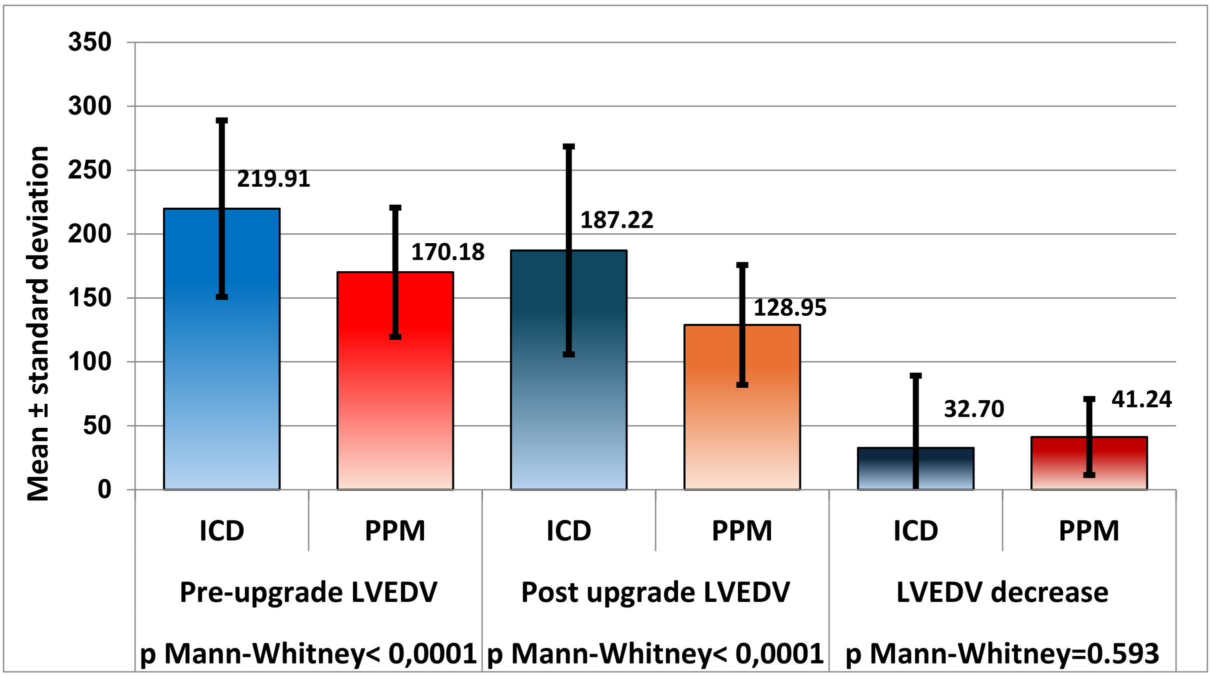

Figure S5: LVEDV was significantly greater in patients with ICD, both pre- and post-upgrade ($p<0.001$). Post upgrade, the PPM group had a greater reduction, however statistically not significant when compared to the ICD group (p Mann-Whitney $p=0.593$).

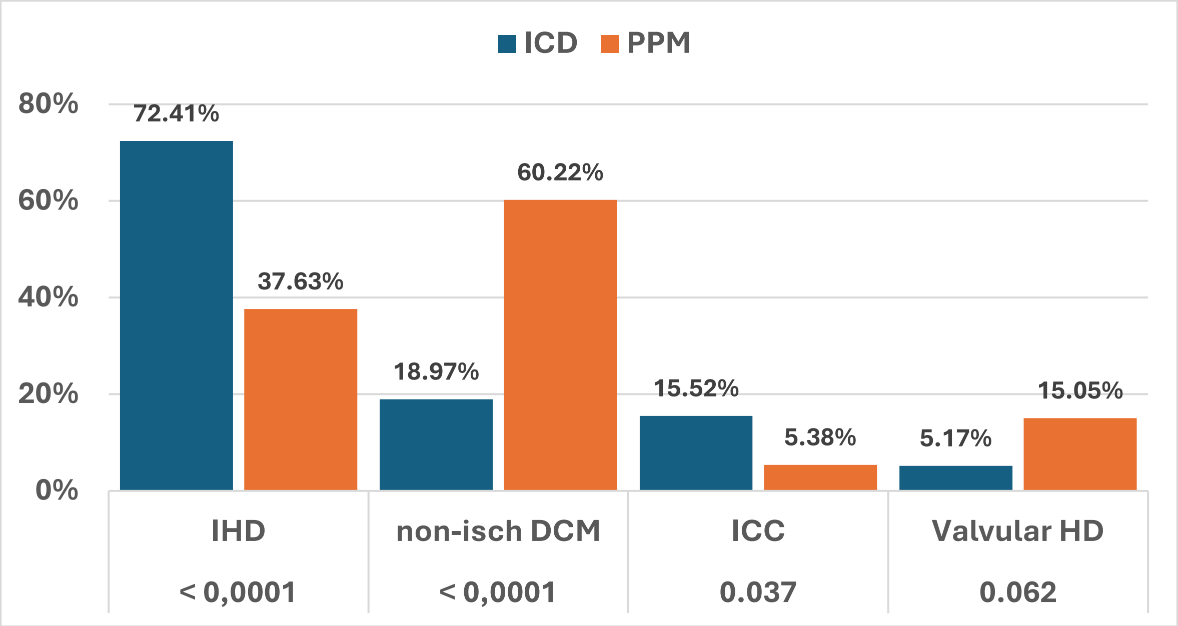

Figure S6: High prevalence of IHD in the ICD group (p Chi square<0,001). High prevalence of non-ischemic DCM in PPM patients.

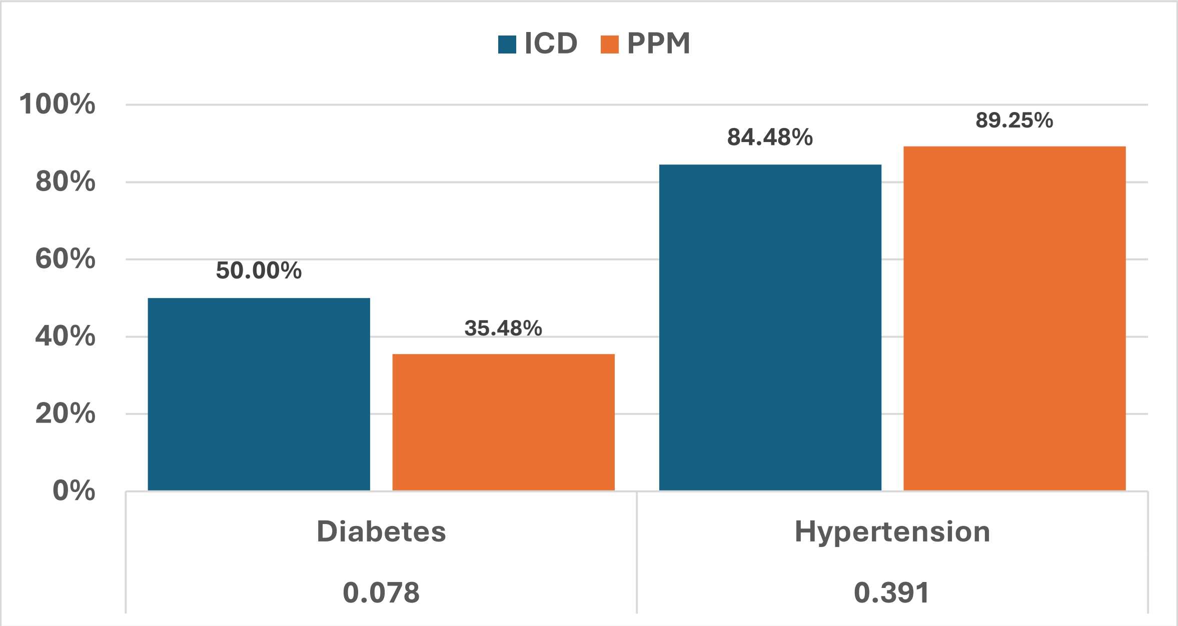

Figure S7: Patients within the ICD group had an increased prevalence of diabetes compared to the PPM group; however, the difference was not statistically significant (p Chi square=0.078>0.05). Hypertension prevalence rates were similar in the two subgroups.

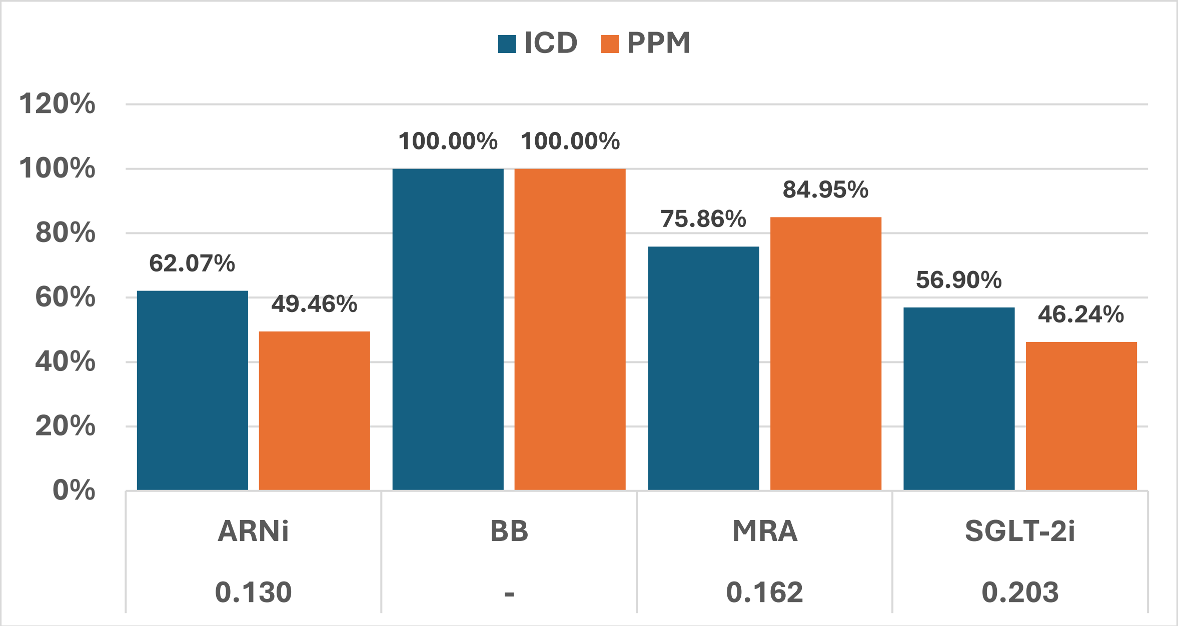

Figure S8: There were no significant differences in the use of the four pillars of heart failure treatment” between the ICD and PPM groups.

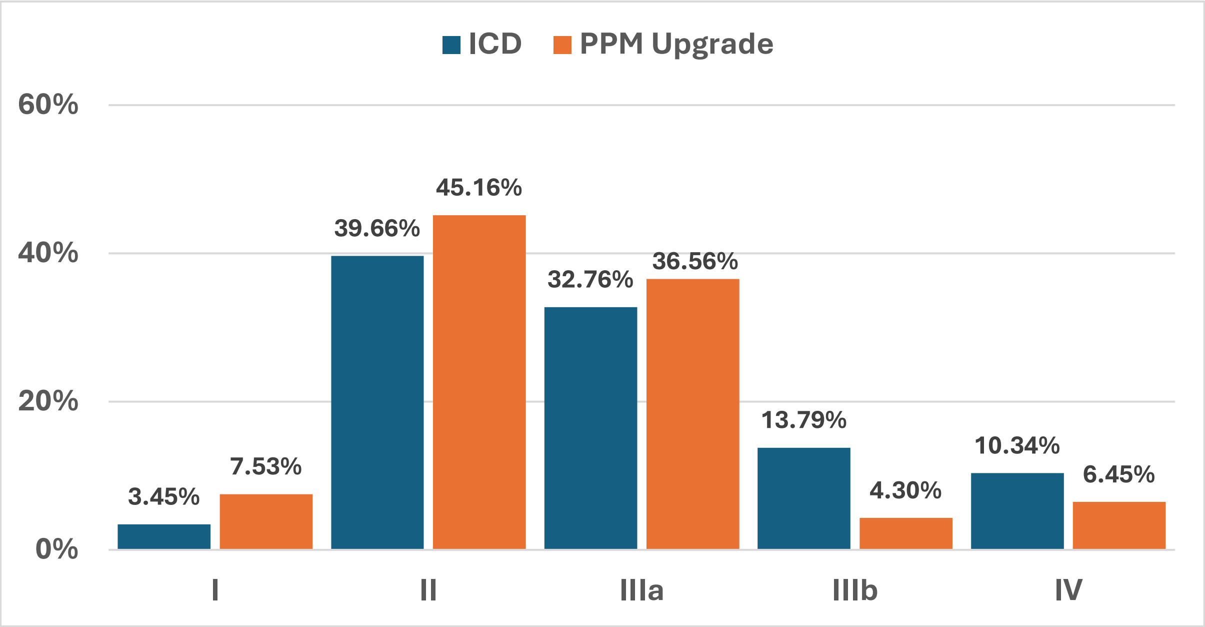

Figure S9: The prevalence of chronic kidney disease within the two subgroups were non-significant (p Chi square=0.189>0.05).
